# Evaluation of the effect of refined management of prospective prescription review rules for antimicrobial agents in an outpatient setting of a county-level hospital in China

**DOI:** 10.1371/journal.pone.0345098

**Published:** 2026-05-21

**Authors:** Dong Pan, Zhewen Lu, Ye Yan

**Affiliations:** Department of Pharmacy, Yangming Hospital Affiliated to Ningbo University (Yuyao People’s Hospital), Yuyao, Zhejiang, China; Fayetteville State University, UNITED STATES OF AMERICA

## Abstract

**Objective:**

To explore the impact of implementing a refined management system for prospective prescription review and to provide evidence for improving the rational use of antibiotics in county-level hospitals.

**Methods:**

Using the prospective prescription review system at Affiliated Yangming Hospital of Ningbo University, China, we performed a refined management of the review rules targeting major issues identified in outpatient antibiotic prescriptions. A comparative analysis was conducted to evaluate the rationality of prescriptions before and after the implementation of this refined management.

**Results:**

Comparison of prescription data before and after the refined management showed that the rate of irrational outpatient antibiotic prescriptions decreased from 20.46% to 7.80%. Specifically, the proportion of prescriptions with inappropriate indications decreased from 2.65% to 0.67%. The rate of prescriptions with an inappropriate frequency of administration or unsuitable solvent decreased from 4.63% to 0.79%. Prescriptions with an inappropriate dosage or route decreased from 8.14% to 2.09%. Irrational prescriptions for special populations decreased from 1.36% to 1.21%, and those involving potential drug-drug interactions decreased from 3.68% to 3.03%.

**Conclusion:**

The refined management of prospective antibiotic prescription review rules in the outpatient department of this county-level hospital significantly reduced the rate of irrational prescriptions. This strategy is worthy of promotion and application in other similar hospitals.

## 1. Introduction

Antimicrobial agents represent a major milestone in modern medicine. The World Health Organization (WHO) clearly defines them as a general term encompassing antibacterial, antiviral, antifungal, and antiparasitic drugs. The advent of these drugs has significantly advanced the treatment of infectious diseases and saved countless lives [1]. However, the global overuse and misuse of these drugs have led to a critical global public health crisis: antimicrobial resistance (AMR). According to the WHO’s 2025 Global Report on Antimicrobial Resistance, more than 1.27 million people die annually from AMR-related infections worldwide. Without effective intervention measures, this number may exceed 10 million by 2050, and AMR will also cause an annual loss of over 10 trillion US dollars to the global economy [1,2]. This issue not only seriously threatens the efficacy of existing treatment regimens but also imposes a substantial burden on healthcare systems worldwide [3,4].

To address the global AMR crisis, the WHO updated the Global Action Plan on Antimicrobial Resistance in 2021, clearly proposing the core goal of “strengthening the management of rational drug use and promoting prescription review interventions based on digital tools.” Many countries and regions have advanced the construction of antimicrobial stewardship systems accordingly [5].

In China, the irrational use of antimicrobial agents has long been a persistent problem, characterized by inappropriate indications, inadequate dosages, and unreasonable treatment durations. This not only increases the risk of adverse drug reactions but also accelerates the emergence of drug-resistant pathogens. To respond to the WHO’s call and standardize clinical drug use, prospective prescription review (PPR) systems have been gradually implemented in many hospitals in China. Previous domestic studies have confirmed that these systems can effectively reduce prescription errors and improve medication safety [6,7]. However, the effectiveness of such systems largely depends on the quality and specificity of their review rules. Generic or poorly calibrated review rules may fail to accurately identify the unique prescribing patterns and emerging irrational drug use trends of different medical institutions, thereby limiting the application value of the systems [8].

Currently, most studies on antimicrobial stewardship and PPR have focused on large medical centers in developed European and American countries and tertiary hospitals in China. There is a relative paucity of detailed studies on the implementation and optimization of such systems in county-level hospitals. As the “foundation” of China’s medical service system, county-level hospitals serve a large population and often face special challenges such as limited resources, unique prescribing habits, and uneven awareness of antimicrobial agents among medical staff [9]. In addition, although the popularity of PPR systems is increasing, there are few research reports on the “refined management” model that continuously optimizes review rules based on the analysis results of in-hospital prescription data, especially in the field of outpatient antimicrobial use management.

Affiliated Yangming Hospital of Ningbo University is a typical county-level hospital in the coastal region of China. During the routine special review of antimicrobial prescriptions, the hospital frequently identified various common problems, including medication without appropriate indications, unreasonable dosage or route, inappropriate frequency of administration or solvent selection, irrational use in special populations, and potential drug-drug interaction risks. To systematically address the above specific problems and respond to the WHO’s initiative of “promoting refined management of antimicrobial agents in primary medical institutions,” this study constructed a refined management strategy by configuring and optimizing targeted review rules based on the hospital’s existing PPR system. This strategy aims to break through the limitations of the traditional general early warning model and create a more precise, data-driven medication intervention tool. Therefore, the main purpose of this study is to evaluate the impact of this refined rule management model on improving the rationality of outpatient antimicrobial prescriptions in county-level hospitals, and to provide Chinese practical reference for the digital management of antimicrobial agents in primary medical institutions worldwide.

## 2. Methods

### 2.1. Study design, data source, and sampling method

This was a pre-post intervention study conducted in the outpatient department of Affiliated Yangming Hospital of Ningbo University, a typical county-level hospital in coastal China. The study aimed to evaluate the impact of refined prospective prescription review (PPR) rules on the rational use of antimicrobial agents.

#### 2.1.1. Data source.

Data were extracted on July 7, 2025, from the hospital’s Prospective Prescription Review System (V6.0), covering the period from July 1, 2024, to June 30, 2025. A total of 348,646 outpatient antimicrobial prescriptions were issued during this one-year period, including 162,761 prescriptions in the pre-intervention period (July 1-December 31,2024) and 185,885 prescriptions in the post-intervention period (January 1-June 30, 2025).

#### 2.1.2. Inclusion and exclusion criteria.

Included antimicrobials adhered to the Guidelines for the Clinical Application of Antimicrobial Agents [10]; excluded drugs were anti-tuberculosis, anti-parasitic, and antiviral agents (Table 1).

**Table 1:** Directory of Commonly Used Antimicrobial Agents in Our Hospital.

Drug Name (Non-proprietary)	Dosage Form and Strength	
	Oral Formulations	Parenteral Formulations
Penicillin	–	Lyophilized powder for injection (80 IU)
Benzathine Benzylpenicillin	–	Lyophilized powder for injection (120 IU)
Cefdinir	Capsule (0.1 g)	–
Cefodizime	–	Lyophilized powder for injection (0.5 g)
Cefuroxime	Tablet (0.25 g)	Lyophilized powder for injection (0.75 g)
Cefaclor	Capsule (0.25 g)	–
	Dry suspension (0.125 g)	–
Cefotiam	Capsule (100 mg)	–
	Granules (50 mg)	–
Cefradine	Capsule (0.25 g)	–
Cefmetazole	–	Lyophilized powder for injection (0.5 g)
Ceftriaxone	–	Lyophilized powder for injection (1.0 g)
Cefoperazone	–	Lyophilized powder for injection (0.5 g)
Latamoxef	–	Lyophilized powder for injection (0.5 g)
Amikacin	–	Injection (0.2 g)
Gentamicin	Dry suspension (0.1 g)	Lyophilized powder for injection (0.5 g)
	Tablet (0.25 g)	–
Clarithromycin	Tablet (0.25 g)	–
Furazolidone	Tablet (0.1 g)	–
Metronidazole	Tablet (0.2 g)	Injection (0.5 g)
Clindamycin Palmitate	–	Injection (0.3 g)
Ornidazole	–	Injection (0.5 g)
Levofloxacin	Tablet (0.5 g)	Injection (0.5 g)
Moxifloxacin	Tablet (0.4 g)	Injection (0.4 g)
Doxycycline	Tablet (0.1 g)	–
Tigecycline	–	Lyophilized powder for injection (50 mg)
Meropenem	–	Lyophilized powder for injection (0.5 g)
Vancomycin	–	Lyophilized powder for injection (0.5 g)
Voriconazole	Tablet (0.2 g)	Lyophilized powder for injection (0.2 g)
Fluconazole	Capsule (50 mg)	Injection (0.2 g)
Itraconazole	Capsule (0.1 g)	–

Note: “–” indicates that the corresponding dosage form is not available in the hospital’s inventory. IU, International Unit.

#### 2.1.3. Sampling strategy.

To ensure statistical robustness, minimize selection bias, and meet repeatability requirements, a systematic random sampling method was adopted. The sample size was calculated using the formula for estimating a population proportion:n = Z^2^π(1 − π)/d^2^, where Z = 1.96 (95% confidence level), π = 20.46% (pre-intervention irrational prescription rate), and d = 2% (margin of error). The minimum required sample size was 1,563 cases per group; actual sampling exceeded this to ensure statistical power.

**Fig 1:**
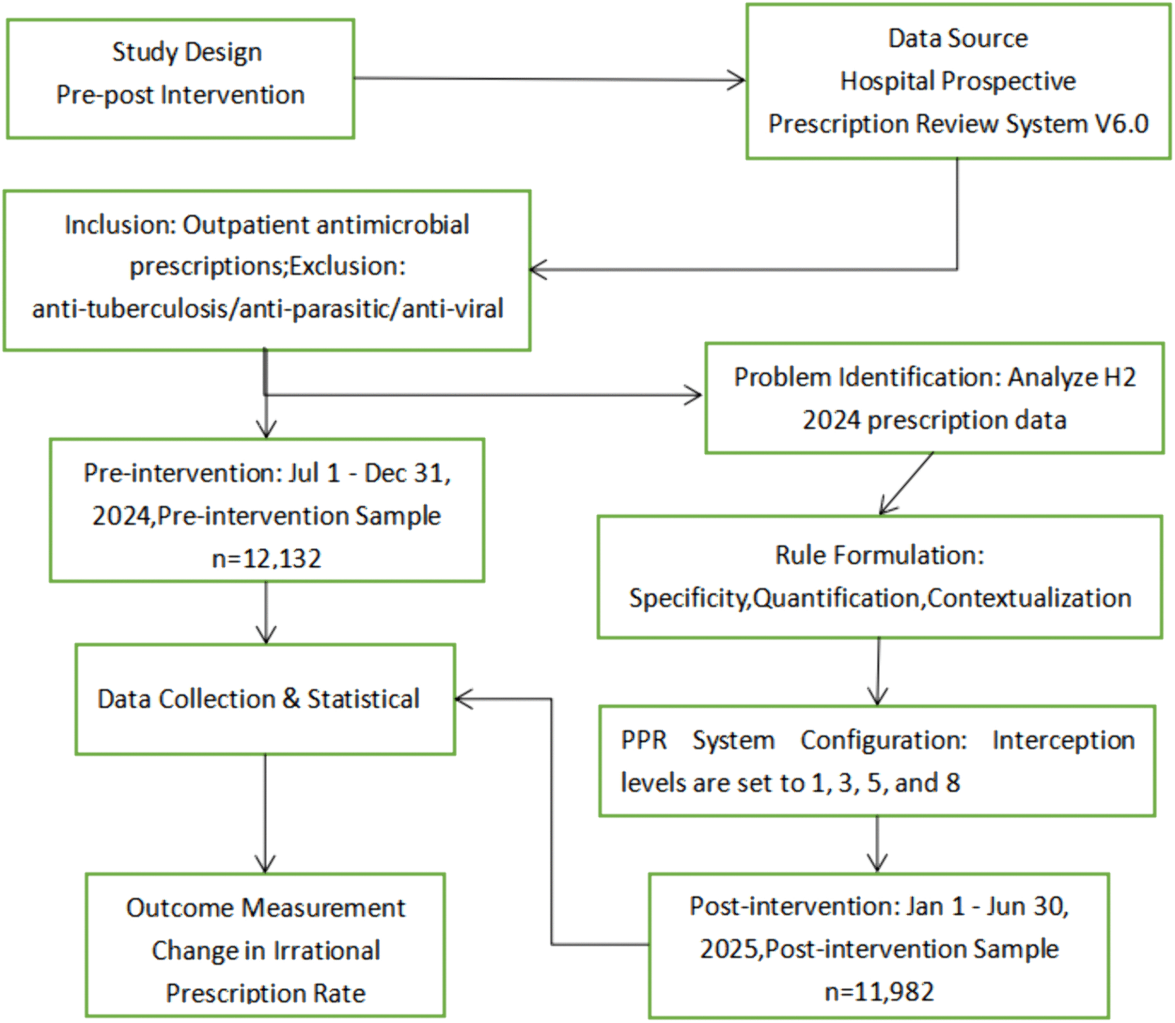
Flow diagram of the study design and sample selection. Data source: Hospital Prospective Prescription Review System (V6.0); study design: Pre-post intervention. Included: Outpatient antimicrobial prescriptions; excluded: Anti-tuberculosis, anti-parasitic, and anti-viral drugs. Sampling method: Systematic random sampling. Pre-intervention period: July 1–December 31, 2024 (n = 12,132); Post-intervention period: January 1–June 30, 2025 (n = 11,982). Core processes: Refined rule formulation → PPR system configuration → Irrational prescription rate analysis.

Sampling steps: Pre-intervention group: Sampling interval k1 = 162,761/12,132 ≈ 13; a random starting point (3) was selected via a random number table (range: 1–k₁); every 13th prescription was selected sequentially from the 3rd prescription until 12,132 valid samples were obtained.Post-intervention group: Sampling interval k2 = 185,885/11,982 ≈ 15; random starting point = 2; 11,982 valid samples were selected using the same sequential method. The overall study workflow is illustrated in Fig 1.

### 2.2. Intervention measures

The core intervention was refined management of prospective antimicrobial prescription review rules, implemented based on the hospital’s existing PPR system (V6.0) and tailored to local high-frequency irrational prescribing issues. All intervention-related details (technical support, alert mechanisms, rule design, and implementation process) are described below:

#### 2.2.1. Technical infrastructure.

The hospital’s “Lianzhong Hospital Information System (HIS) V5.0” was fully interfaced with the “Prospective Prescription Review System (PPR) V6.0” to enable real-time data synchronization and rule execution: After a physician prescribes and saves an order in HIS, key data (diagnosis, drug name, dosage, route of administration, patient demographics, laboratory results, historical medication records) are automatically synchronized to PPR V6.0. The PPR system conducts real-time audits with a response time ≤ 5 seconds. For prescriptions involving special populations or potential drug-drug interactions, the PPR system retrieves additional data from HIS (e.g., liver function test results, previous medication history) to support accurate judgment. Review rules can be adjusted in real time, with modification logs automatically recorded for traceability.

#### 2.2.2. Prescription review alert levels.

**Fig 2:**
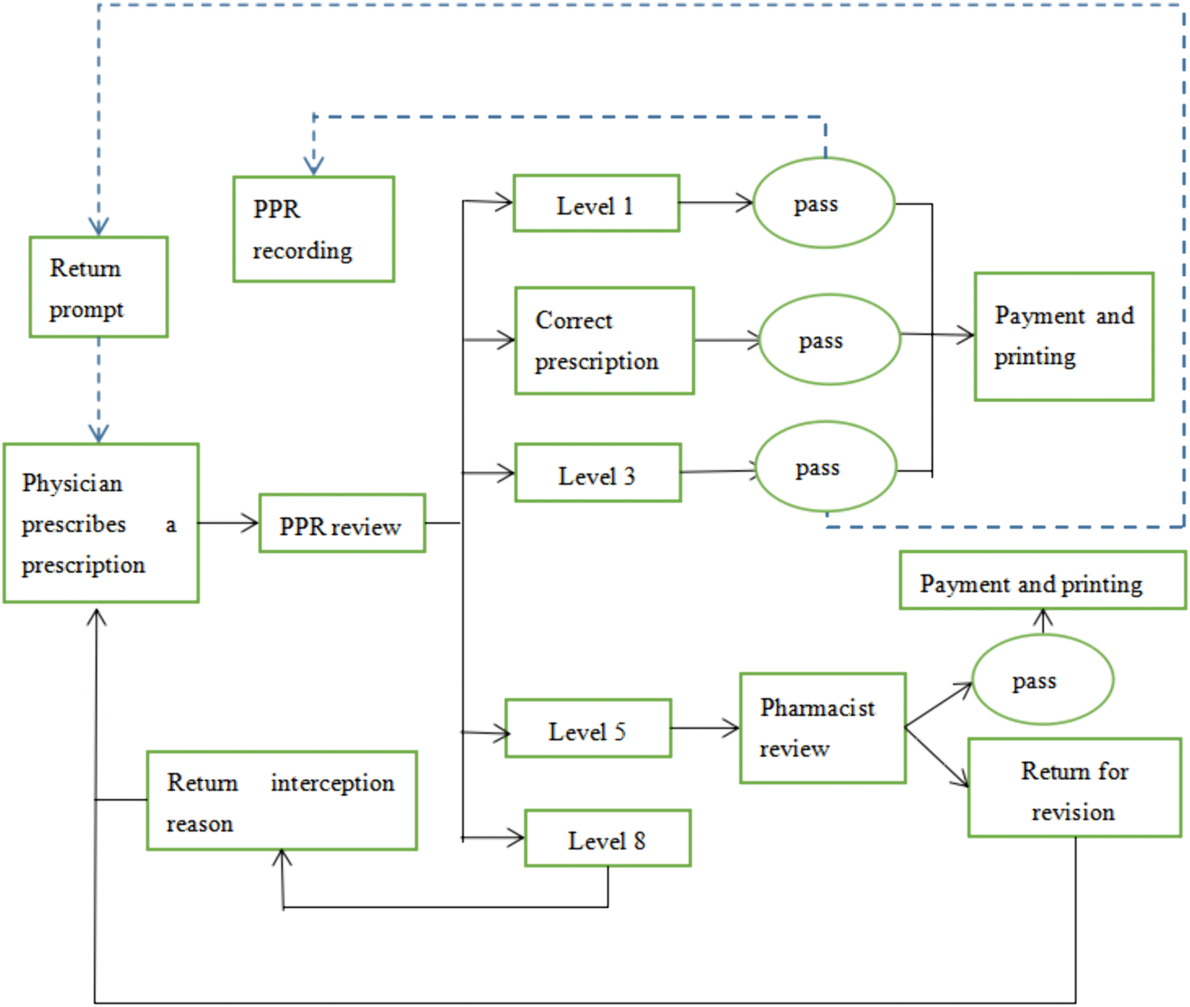
Flow diagram of prescription processing in the prospective prescription review system. Physicians prescribe → PPR system conducts real-time review, processed by alert levels: Level 1 (Acceptable): Record and release; level 3 (Use with caution): Pop-up reminder; level 5 (Not recommended): Intercept for pharmacist review; level 8 (Contraindicated): Mandatory block, resubmit after modification.

The system adopts a non-linear alert classification (Levels 1, 3, 5, 8) to avoid redundant ambiguity, with automatic alerts as the primary method and pharmacist manual intervention as a supplement (Table 2). The workflow for handling alerts is as follows (Fig 2): Level 8 (Contraindicated): Mandatory prescription block; physicians must modify the prescription without appeal. Level 5 (Not recommended): Prescription interception; physicians must provide justification for pharmacist re-review. Level 3 (Use with caution): No interception; pop-up reminder for physicians. Level 1 (Acceptable): No interception; only recorded in pharmacists’ backend logs. For Level 5 alerts, a predefined 30-second judgment time is allowed to ensure workflow efficiency.

**Table 2:** Alert Levels in the Prescription Pre-review System.

Alert Level	Definition	System Intervention
Level 8	Contraindicated. Extremely high-risk issues, including methods explicitly contraindicated in the drug package insert or violations of current prescribing regulations.	The prescription is blocked mandatorily. The physician cannot appeal and must modify the prescription.
Level 5	Not recommended. Potential medication risks exist, requiring re-confirmation by the prescribing physician and pharmacist.	The prescription is intercepted. The physician must provide an appropriate justification on the computer terminal for re-review by the pharmacist.
Level 3	Use with caution. Relatively low medication risk, but monitoring should be intensified during use.	Not intercepted. A pop-up reminder is displayed to alert the physician.
Level 1	Acceptable. Minimal medication risk.	Not intercepted. The interaction is only recorded in the pharmacist’s backend log.

#### 2.2.3. Refined prescription review rules.

Based on hospital-specific irrational prescribing patterns (identified via pre-intervention prescription audits) and national/international guidelines, 63 targeted review rules were developed. The rules cover five core categories (S1–S5 Tables):

Indication rules: Require clear evidence of bacterial infection (e.g., positive pathogen culture); flag prescriptions for viral infections (e.g., viral cold) or off-label use (e.g., moxifloxacin for urinary tract infections). Frequency of administration and solvent rules: Prohibit non-compliance with package inserts (e.g., ceftriaxone ≤ once daily) and incorrect solvent volumes leading to improper concentrations. Dosage and route rules: Enforce package insert specifications for single/daily doses and dosing route (e.g., intravenous benzathine penicillin); require weight-based pediatric dosing (e.g., azithromycin suspension 5–10 mg/kg for minors). Special population rules: Stratify populations (minors: < 8/ < 14/ < 18 years; pregnant/lactating women; hepatic impairment) and define contraindications (e.g., moxifloxacin in minors/pregnant women), dosage restrictions (e.g., vancomycin ≤4 g/day for <14 years), and monitoring requirements (e.g., cephalosporins in pregnant women).

Drug-drug interaction rules: Target 8 high-risk drug pairs (e.g., meropenem + sodium valproate, ceftriaxone + calcium gluconate) based on package inserts and Medication Assistant V15.7.

#### 2.2.4. Rule formulation and implementation process.

Formulation subject: A cross-departmental working group was established, including pharmacists (rule drafting/evidence verification), clinical experts (Infectious Diseases, Respiratory Medicine, Pediatrics), IT engineers (system configuration/data docking), and medical administrators (rule approval/dispute resolution). Key improvements over old rules: Compared to the original general rules, the refined rules are hospital-specific, dynamically optimized monthly (Table 3).Implementation timeline: The refined rules were configured in PPR V6.0 in December 2024 and officially launched on January 1, 2025 (start of the post-intervention period).

**Table 3:** Differences Between New and Old Rules.

Aspect	Old Rules	New Refined Rules
Targeting	General (applicable to all hospitals)	Hospital-specific (focused on 5 local high-frequency irrational issues)
Special population coverage	Basic categories only	Detailed stratification (e.g., minors divided into < 8 years/ < 14 years/ < 18 years)
Flexibility	Fixed, non-adjustable	Dynamic (monthly optimization based on real-world data)

### 2.3. Statistical analysis

Statistical analyses were performed using IBM SPSS Statistics version 27. Variable presentation: Categorical variables (e.g., irrational prescription counts/rates) are reported as absolute numbers (n) and percentages, with 95% confidence intervals (CIs) for rates.Group comparisons: The Chi-square test was used to compare irrational prescription rates (overall and by category) between pre- and post-intervention groups. Effect quantification: Two metrics were calculated to assess practical significance beyond statistical significance (P < 0.05): Risk Difference (RD) with 95% CI: Absolute reduction in irrational prescription rates.Cohen’s w: Effect size (small = 0.1, moderate = 0.3, large = 0.5).

### 2.4. Reference materials

The following materials guided rule development and prescription review:Hospital prescription review reports (July–December 2024); Drug package inserts; Guidelines for the Clinical Application of Antimicrobial Agents [10], Hospital Prescription Review Management Norms [11], Prescription Management Measures [12]; Clinical tools: Medication Assistant V15.7.

### 2.5. Ethics approval

The study was conducted in accordance with ethical standards and approved by the Institutional Review Board of Affiliated Yangming Hospital of Ningbo University (Approval No. AF/SC-11/01.0).

## 3. Results

**Fig 3:**
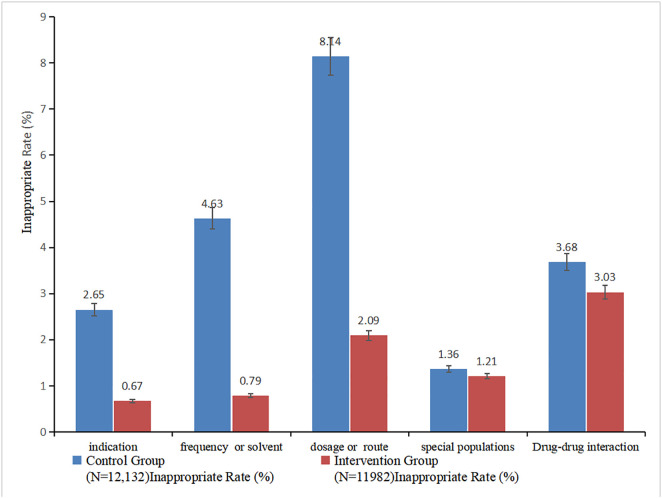
Comparison of inappropriate outpatient antimicrobial prescription rates across different categories before and after intervention. X-axis: Categories of irrational prescriptions (indication, special populations, drug-drug interaction, dosage/route, administration frequency/solvent); Y-axis: irrational prescription rate (%). Rates of control group (N = 12,132) vs. intervention group (N = 11,982): 2.65%/0.67%, 1.36%/1.21%, 3.68%/3.03%, 8.14%/2.09%, 4.63%/0.79%.

The refined management of prescription review rules significantly improved the rationality of outpatient antimicrobial prescribing. Analysis of 12,132 pre-intervention and 11,982 post-intervention prescriptions showed that the overall rate of irrational prescriptions decreased substantially from 20.46% to 7.80%, with an absolute reduction of 12.66 percentage points (P < 0.01, Cohen’s w = 0.18), indicating a small-to-moderate intervention effect (Table 4). Marked improvements were observed across most key categories of irrational prescribing (Fig 3, Table 4):

**Table 4:** Comparison of Types of irrational Antimicrobial Prescriptions.

Review Category	Control Group (N = 12,132)		Intervention Group (N = 11,982)		Statistical Analysis		
	n (irrational)	Rate (%)	n (irrational)	Rate (%)	P	RD (95%CI)	Effect Size (Cohen’s w)
Inappropriate indication	321	2.65	80	0.67	<0.01	−1.98% (−2.23, −1.73)	0.08
Inappropriate frequency/solvent	562	4.63	95	0.79	<0.01	−3.84% (−4.18, −3.50)	0.12
Inappropriate dosage/route	988	8.14	251	2.09	<0.01	−6.05% (−6.46, −5.64)	0.14
Inappropriate use in special populations	165	1.36	145	1.21	>0.05	−0.15% (−0.39, 0.09)	0.01
Potential drug-drug interaction	446	3.68	363	3.03	<0.01	−0.65% (−1.04, −0.26)	0.02
Total	2482	20.46	934	7.80	<0.01	−12.66% (−13.42, −11.90)	0.18

Note: χ² values for each category are as follows: inappropriate indication (144.27), inappropriate frequency/solvent (335.31), inappropriate dosage/route (452.43), inappropriate use in special populations (1.07), potential drug-drug interaction (7.77).The pooled χ² value for the total irrational prescription rate was 794.70; RD = Risk Difference; CI = Confidence Interval.

Inappropriate indications: Declined from 2.65% to 0.67% (P < 0.01), reflecting a significant reduction in antimicrobial use without evidence of bacterial infection or off-label prescribing.Inappropriate frequency of administration or solvent: Fell sharply from 4.63% to 0.79% (P < 0.01), addressing non-compliance with package inserts and incorrect solvent selection.Inappropriate dosage or route: The most prominent improvement, dropping from 8.14% to 2.09% (P < 0.01), correcting deviations in single/daily doses, dosing route, and pediatric weight-based prescribing.Potential drug-drug interactions: A modest but statistically significant decrease was noted, from 3.68% to 3.03% (P< 0.01). In contrast, the rate of inappropriate use in special populations (pregnant/lactating women, minors, patients with hepatic impairment) showed no statistically significant change, with a slight reduction from 1.36% to 1.21% (P> 0.05, Cohen’s w = 0.01), indicating minimal practical impact of the intervention on this subgroup.Detailed statistical parameters (risk difference, 95% confidence intervals, and χ² values) for each category are provided in Table 4, and the comparative distribution of irrational prescription rates is visualized in Fig 3.

## 4. Discussion

This study focuses on the practical application of refined prospective prescription review rules in county-level hospitals, a key gap in current antimicrobial stewardship research. The core finding—significant improvement in rational antimicrobial prescribing—reflects the targeted effectiveness of the intervention, which addresses the unique challenges of primary medical institutions.

### 4.1. Mechanisms underlying the effectiveness of refined management

The substantial reduction in overall irrational prescription rates confirms that refined review rules are far more effective than generic ones for county-level hospitals. Unlike the default general rules, the optimized rules were tailored to the hospital’s high-frequency irrational issues and integrated real-time data synchronization between HIS and PPR systems. Real-time alerts (≤5 seconds response time) and hierarchical intervention strategies (Levels 1/3/5/8) enabled physicians to correct errors promptly, while minimizing unnecessary disruptions to clinical workflows. This aligns with the WHO’s emphasis on “digital tool-based prescription review”—the combination of hospital-specific rules and efficient technical support overcomes the limitations of one-size-fits-all systems, making antimicrobial stewardship feasible in resource-constrained primary care settings.

### 4.2. Why special populations showed no significant improvement

The lack of statistically significant reduction in irrational prescriptions for special populations (pregnant/lactating women, minors, patients with hepatic impairment) requires in-depth contextual analysis. First, the baseline irrational rate for this subgroup was already low (1.36%), reflecting clinicians’ inherent caution when prescribing for high-risk groups—this “ceiling effect” limits further improvements. Second, special population prescribing involves complex clinical judgments (e.g., balancing maternal benefits and fetal risks, adjusting doses for hepatic function), which cannot be fully addressed by rule-based alerts alone. Although the refined rules stratified minors into three age groups and added contraindications for severe hepatic impairment, some nuanced clinical scenarios (e.g., off-label use for refractory infections) still rely on physician expertise, leading to limited intervention impact. Third, the relatively small sample size of this subgroup may have reduced statistical power to detect subtle changes, which should be verified in larger-scale multi-center studies.

### 4.3. Clinical and public health significance for county-level healthcare

The intervention’s clinical value extends beyond numerical reductions in irrational prescriptions. County-level hospitals serve as the primary healthcare provider for rural and suburban populations, handling large patient volumes—even a modest reduction in inappropriate prescribing translates to widespread improvements in medication safety. For example, the marked decline in dosage/route errors and inappropriate administration routes directly reduces the risk of adverse drug events. More importantly, reducing unnecessary antimicrobial use alleviates selective pressure on pathogens, slowing the emergence of antimicrobial resistance (AMR) in communities. This aligns with the WHO’s global action plan to combat AMR, as primary care institutions are key frontlines for curbing inappropriate drug use. For resource-limited county-level hospitals, this low-cost, technology-driven intervention (based on existing PPR systems) offers a sustainable model to balance clinical needs and public health goals.

### 4.4. Key factors for successful implementation: Role transformation and multi-departmental collaboration

The success of refined management hinges on two critical pillars: the transformation of pharmacists’roles and robust multi-departmental collaboration. Traditional prescription review focuses on passive error correction, but the refined model requires pharmacists to act as “data analysts” and “rule optimizers”—they must continuously extract insights from prescription data to update rules monthly, verify evidence, and communicate with physicians to resolve complex alerts. This role expansion addresses the long-standing issue of uneven antimicrobial awareness among medical staff in county-level hospitals, fostering a culture of evidence-based prescribing. Additionally, cross-departmental collaboration ensures intervention feasibility: clinical experts from Infectious Diseases, Pediatrics, and Respiratory Medicine provided practical insights to avoid over-constraining clinical practice; IT engineers guaranteed seamless data docking between HIS and PPR systems; and medical administrators resolved disputes to promote rule adherence. Without this collaborative framework, even well-designed rules would struggle to be implemented effectively.

### 4.5. Limitations

It is important to acknowledge the limitations of this study. The rule set was optimized based on known local prescribing issues and thus may not encompass all potential errors, particularly less common drug interactions. The initial identification of unanticipated irrational prescriptions remains dependent on pharmacist vigilance. Finally, as a single-center study, the generalizability of our findings to other hospitals with different patient demographics, prescribing cultures, or information system capabilities may be limited.

## 5. Conclusion

The refined management of prospective antimicrobial prescription review rules in this county-level hospital’s outpatient department significantly reduced the overall irrational prescription rate—from 20.46% to 7.80%—with marked improvements in inappropriate indications, dosage/route, and frequency of administration/solvent use.

Beyond confirming the intervention’s effectiveness, this work carries broader significance for addressing global antimicrobial resistance (AMR) and optimizing primary healthcare. As the cornerstone of China’s medical system, county-level hospitals face unique resource constraints and prescribing challenges, yet our findings demonstrate that data-driven, locally tailored review rules can overcome these barriers—aligning with the WHO’s Global Action Plan for AMR and filling a critical research gap in primary care stewardship, particularly for low- and middle-income countries.

Practically, this model offers a low-cost, replicable solution for similar institutions: leveraging existing hospital information systems (HIS) and prospective prescription review (PPR) platforms, prioritizing local prescription data to target high-frequency irrational issues, and relying on cross-departmental collaboration to balance rigor and clinical feasibility. Dynamic rule optimization further ensures adaptability to emerging prescribing trends.

In summary, this refined management strategy not only enhances medication safety for rural and suburban populations but also provides actionable evidence for scaling precision antimicrobial stewardship across primary care settings. Its promotion has the potential to mitigate AMR at the community level and elevate global primary healthcare quality.

## Supporting information

S1 Table Rules for Indication Settings of Antimicrobial Agents in the Prescription Pre-review System V6.0. (DOCX)

S2 Table Rules for Administration Frequency and Solution Concentration Settings of Antimicrobial Agents in the Prescription Pre-review System V6.0. (DOCX)

S3 Table Rules for Dosage and Route Settings of Antimicrobial Agents in the Prescription Pre-review System V6.0. (DOCX)

S4 Table Rules for Special Population Settings of Antimicrobial Agents in the Prescription Pre-review System V6.0. (DOCX)

S5 Table Rules for Drug-Drug Interaction Settings of Antimicrobial Agents in the Prescription Pre-review System V6.0. (DOCX)
