# Supplementary material for: Evaluation of the effect of refined management of prospective prescription review rules for antimicrobial agents in an outpatient setting of a county-level hospital in China
Source: PLoS One. 2026 May 21;21(5):e0345098. doi: 10.1371/journal.pone.0345098 (PMC13193398; doi:10.1371/journal.pone.0345098)
Supplement: S1 Table — (DOCX) [file pone.0345098.s001.docx]

S1 Table. Rules for Indication Settings of Antimicrobial Agents in the Prescription Pre-review System V6.0

| Indication | Indication | Alert Level | Warning Message |
| --- | --- | --- | --- |
| Cefuroxime, Cefaclor, Cefixime, Cefradine, Cefmetazole, Ceftriaxone, Cefoperazone, Latamoxef, Amikacin, Gentamicin, Azithromycin, Clarithromycin, Metronidazole, Clindamycin, Ornidazole, Doxycycline, Tigecycline, Meropenem, Vancomycin, Voriconazole, Fluconazole, Itraconazole. | Infections caused by susceptible bacteria | 5 | "The diagnosis does not match the drug indication." |
| Moxifloxacin | Urinary tract infections | 5 | "This drug is not recommended for the treatment of urinary tract infections." |

Note: A warning message prompts if the prescription diagnosis does not match the listed indications.
