# Supplementary material for: Evaluation of the effect of refined management of prospective prescription review rules for antimicrobial agents in an outpatient setting of a county-level hospital in China
Source: PLoS One. 2026 May 21;21(5):e0345098. doi: 10.1371/journal.pone.0345098 (PMC13193398; doi:10.1371/journal.pone.0345098)
Supplement: S2 Table — (DOCX) [file pone.0345098.s002.docx]

S2 Table. Rules for Administration Frequency and Solution Concentration Settings of Antimicrobial Agents in the Prescription Pre-review System V6.0

| Drug Name | Dosage Form | Administration Frequency / Solution Concentration | Alert Level | Warning Message |
| --- | --- | --- | --- | --- |
| Clindamycin Palmitate | Injection | < 6 mg/mL | 5 | "The concentration of clindamycin palmitate solution should not exceed 6 mg/mL." |
| Tigecycline | Injection | < 1 mg/mL | 5 | "The concentration of tigecycline solution should not exceed 1 mg/mL." |
| Cefodizime | Injection | 0.125 - 0.25 g/mL | 5 | "The recommended concentration for cefodizime solution is 0.125 - 0.25 g/mL." |
| Voriconazole | Injection | 0.5 - 5 mg/mL | 5 | "The recommended concentration for voriconazole solution is 0.5 - 5 mg/mL." |
| Meropenem | Injection | < 50 mg/mL | 5 | "The concentration of meropenem solution should not exceed 50 mg/mL." |
| Amikacin | Injection | QD-BID | 5 | "The administration frequency for amikacin is once or twice daily." |
| Cefixime | Capsule / Granules | BID | 5 | "The administration frequency for cefixime is twice daily." |
| Ceftriaxone | Injection | QD | 5 | "The administration frequency for ceftriaxone is once daily." |
| Vancomycin | Injection | TID-QID | 5 | "The administration frequency for vancomycin is 3-4 times daily." |

Note: A warning message prompts if the prescribed administration frequency or concentration exceeds the above settings.
