# Supplementary material for: Evaluation of the effect of refined management of prospective prescription review rules for antimicrobial agents in an outpatient setting of a county-level hospital in China
Source: PLoS One. 2026 May 21;21(5):e0345098. doi: 10.1371/journal.pone.0345098 (PMC13193398; doi:10.1371/journal.pone.0345098)
Supplement: S3 Table — (DOCX) [file pone.0345098.s003.docx]

S3 Table. Rules for Dosage and Route Settings of Antimicrobial Agents in the Prescription Pre-review System V6.0

| Drug Name | Dosage Form | Dosage / Route | Alert Level | Warning Message |
| --- | --- | --- | --- | --- |
| Azithromycin | Suspension | 5-10 mg/kg QD | 8 | "For patients under 18 years, the single daily dose of azithromycin is 5-10 mg/kg." |
| Fluconazole | Injection | 50-400 mg QD | 5 | "For patients over 18 years, the single daily dose of fluconazole is 50-400 mg." |
| Clarithromycin | Extended-release tablet | 0.5-1.0 g QD | 5 | "For patients over 12 years, the single daily dose of clarithromycin is 0.5-1.0 g." |
| Clindamycin Palmitate | Injection | 0.6-2.7 g QD | 5 | "For patients over 18 years, the single daily dose of clindamycin palmitate is 0.6-2.7 g." |
| Latamoxef | Injection | 40-150 mg/kg BID-QID | 5 | "For patients under 14 years, the daily dose of latamoxef is 40-150 mg/kg, administered in 2-4 divided doses." |
| Tigecycline | Injection | 200 mg QD | 5 | "For patients over 18 years, the single daily dose of tigecycline should not exceed 200 mg." |
| Cefdinir | Capsule | 0.1 g TID | 5 | "For patients over 18 years, the total daily dose of cefdinir is 0.3 g." |
| Cefuroxime | Injection | 1.5-3.0 g QD | 5 | "For patients over 18 years, the single daily dose of cefuroxime is 1.5-3.0 g." |
| Cefmetazole | Injection | 1-4 g QD | 5 | "For patients over 18 years, the single daily dose of cefmetazole is 1-4 g." |
| Levofloxacin | Tablet / Injection | 0.25-0.75 g QD | 8 | "For patients over 18 years, the single daily dose of levofloxacin is 0.25-0.75 g." |
| Cefoperazone | Injection | 0.5-4 g QD | 5 | "For patients over 18 years, the single daily dose of cefoperazone is 0.5-4 g." |
| Benzathine Benzylpenicillin | Injection | Intramuscular injection | 5 | "The package insert for benzathine benzylpenicillin does not specify administration routes other than intramuscular injection." |
| Penicillin | Injection | Intramuscular injection / Intravenous drip | 5 | "The package insert for penicillin does not specify administration routes other than intramuscular injection or intravenous drip." |

Note: A warning message prompts if the prescribed dosage or route exceeds the above settings. QD, once daily; BID, twice daily; TID, three times daily; QID, four times daily.
