# Supplementary material for: Evaluation of the effect of refined management of prospective prescription review rules for antimicrobial agents in an outpatient setting of a county-level hospital in China
Source: PLoS One. 2026 May 21;21(5):e0345098. doi: 10.1371/journal.pone.0345098 (PMC13193398; doi:10.1371/journal.pone.0345098)
Supplement: S4 Table — (DOCX) [file pone.0345098.s004.docx]

S4 Table. Rules for Special Population Settings of Antimicrobial Agents in the Prescription Pre-review System V6.0

| Drug Name | Dosage Form | Special Population | Alert Level | Warning Message |
| --- | --- | --- | --- | --- |
| Cefuroxime, Cefaclor, Cefixime, Cefradine, Cefmetazole, Ceftriaxone, Cefoperazone | Capsule/  Tablet / Injection | Pregnant and lactating women | 3 | "Use with caution in pregnant women." |
| Moxifloxacin | Tablet / Injection | Pregnant and lactating women | 8 | "Moxifloxacin is contraindicated in pregnant and lactating women." |
|  |  | Severe hepatic impairment* | 8 | "Moxifloxacin is contraindicated in patients with severe hepatic impairment." |
|  |  | Under 18 years | 8 | "Moxifloxacin is contraindicated in patients under 18 years of age." |
| Levofloxacin | Tablet / Injection | Pregnant and lactating women | 8 | "Levofloxacin is contraindicated in pregnant and lactating women." |
|  |  | Under 18 years | 8 | "Levofloxacin is contraindicated in patients under 18 years of age." |
| Ornidazole | Injection | Under 18 years | 5 | "Safety and effectiveness of ornidazole in patients under 18 years have not been established." |
| Vancomycin | Injection | Under 14 years | 5 | "The daily dose of vancomycin for patients under 14 years should not exceed 4 g." |
| Doxycycline | Capsule | Under 8 years | 8 | "Doxycycline is contraindicated in children under 8 years of age." |
| Cefixime | Capsule | Difficulty swallowing | 5 | "Cefixime capsules should be used cautiously in patients with swallowing difficulties." |

*Note: A warning message prompts if the prescription violates the above rules for special populations. * Indicators for Severe Hepatic Impairment in the Moxifloxacin Prescribing Information: AST(Aspartate Aminotransferase) or ALT(Alanine Aminotransferase)≥ 200 U/L.*
