# Supplementary material for: Evaluation of the effect of refined management of prospective prescription review rules for antimicrobial agents in an outpatient setting of a county-level hospital in China
Source: PLoS One. 2026 May 21;21(5):e0345098. doi: 10.1371/journal.pone.0345098 (PMC13193398; doi:10.1371/journal.pone.0345098)
Supplement: S5 Table — (DOCX) [file pone.0345098.s005.docx]

S5 Table. Rules for Drug-Drug Interaction Settings of Antimicrobial Agents in the Prescription Pre-review System V6.0

| Primary Drug | Dosage Form | Interacting Drug | Alert Level | Warning Message (Source) |
| --- | --- | --- | --- | --- |
| Meropenem | Injection | Sodium Valproate Injection | 5 | "Package Insert: Concomitant use of meropenem and valproic acid may decrease valproic acid serum concentrations, potentially leading to seizures." |
| Ceftriaxone | Injection | Calcium Gluconate Injection | 5 | "FDA Warning: Ceftriaxone and calcium-containing products should not be mixed due to the risk of serious adverse events." |
| Moxifloxacin | Tablet | Aluminum Phosphate Gel | 3 | "Package Insert: Concomitant administration with drugs containing polyvalent cations may significantly reduce bioavailability. Dosing should be separated by at least 4 hours." |
| Metronidazole | Injection | Hydrocortisone Injection (containing ethanol) | 5 | "Package Insert: Concomitant use of metronidazole with alcohol-containing drugs may cause alcohol accumulation, leading to a disulfiram-like reaction." |
| Clarithromycin | Extended-release tablet | Mizolastine Extended-release tablet | 5 | "Package Insert: Clarithromycin may increase the plasma concentration of mizolastine." |
| Azithromycin | Suspension | Mizolastine Extended-release tablet | 5 | "Package Insert: Azithromycin may increase the plasma concentration of mizolastine." |
| Itraconazole | Capsule | Mizolastine Extended-release tablet | 5 | "Package Insert: Itraconazole may moderately increase the plasma concentration of mizolastine." |
| Furazolidone | Tablet | Carbamazepine Tablet | 5 | "Package Insert: Furazolidone may increase the plasma concentration of carbamazepine." |

Note: A pop-up warning message prompts when the above drug combinations are prescribed.
